# Effect of Korean red ginseng on deep capillary plexus parameters in diabetic retinopathy: A prospective, randomized, double-blind clinical trial

**DOI:** 10.1016/j.jgr.2026.101025

**Published:** 2026-03-31

**Authors:** Dong Hyun Lee, Chul Hee Lee, Tae Young Kim, Junwon Lee, Eun Young Choi, Min Kim

**Affiliations:** a Department of Ophthalmology, Yonsei University College of Medicine, Seoul, Republic of Korea; b Department of Ophthalmology, Myongji Hospital, Goyang, Republic of Korea; c Seoul Eye Center, Gimhae, Republic of Korea

**Keywords:** Deep capillary plexus, Diabetic retinopathy, Microvascular changes, Optical coherence tomography angiography, Korean red ginseng, Vessel length density

## Abstract

**Background:**

This prospective, randomized, double-blind clinical trial aimed to evaluate the effects of Korean Red Ginseng (KRG) extract on the retinal microvascular parameters in patients with diabetic retinopathy (DR).

**Methods:**

Patients with mild to moderate non-proliferative DR were randomized to receive KRG extract or placebo for 90 days. Outcomes included changes in the best-corrected visual acuity (BCVA), intraocular pressure (IOP), central macular thickness (CMT), and optical coherence tomography angiography (OCTA) parameters, including the foveal avascular zone, vessel length density (VLD), and perfusion index (PI) in the superficial and deep capillary plexuses (SCP and DCP, respectively). The incidence of proliferative DR or diabetic macular edema (DME) was also monitored.

**Results:**

Twenty-four patients were enrolled and 23 completed the study. The baseline BCVA, IOP, CMT, and OCTA parameters were comparable between the groups (all p > 0.05). No significant changes were observed in BCVA, CMT, DR severity stage, or incidence of DME over the 3-month follow-up period in either group. Significant group × time interaction effects were observed for inferior DCP VLD (p = 0.011) and for PI in the superior (p = 0.044) and inferior (p = 0.026) DCP regions. No significant interaction effects were identified for SCP parameters or other DCP subfields. No adverse events were reported.

**Conclusions:**

KRG extract was associated with short-term, region-specific changes in OCTA-derived DCP perfusion metrics over 3 months. These findings reflect alterations in OCTA-derived microvascular parameters and do not establish modification of clinical outcomes such as DR progression or the development of DME.

**Trial registration:**

Clinical Research Information Service, #KCT0010991.

## Graphical abstract

**Figure:**
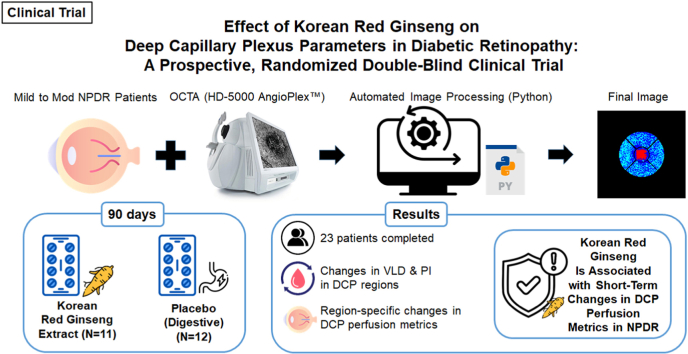

## 1 Introduction

Diabetic retinopathy (DR) is a chronic microvascular complication of diabetes and remains a leading cause of vision impairment worldwide. Persistent hyperglycemia induces oxidative stress, inflammation, and metabolic dysregulation, resulting in endothelial dysfunction, capillary nonperfusion, and progressive retinal ischemia. These pathologic alterations contribute to microvascular dropout and disruption of the blood–retinal barrier, ultimately leading to vision-threatening complications [1]. Diabetic macular edema (DME), one of the most common causes of visual loss in DR, arises from increased vascular permeability and breakdown of retinal vascular integrity under conditions of sustained metabolic stress [2]. Although intravitreal anti–vascular endothelial growth factor (VEGF) agents and corticosteroids constitute the mainstay of DME treatment [3], repeated injections are often required, and a substantial proportion of patients exhibit suboptimal or incomplete responses [4]. These limitations underscore the need for adjunctive or preventive strategies targeting early microvascular dysfunction in DR.

Ginseng refers to the root of plants belonging to the genus *Panax*, and has been widely used as a medicinal herb, in traditional Eastern Asian medicine, particularly in countries such as China and Korea. In modern medicine, efforts to apply the therapeutic potential of ginseng in metabolic and vascular diseases have been underway for many years. Numerous studies have demonstrated the anti-inflammatory and antiangiogenic effects of ginseng as well as its ability to suppress microvascular leakage [5]. Korean Red Ginseng (KRG) has been reported to modulate gene expression profiles associated with diabetic retinal injury in experimental models [6]. In addition, investigations of individual ginsenoside derivatives derived from *Panax* species have shown inhibition of VEGF expression, attenuation of apoptosis, protection against hyperglycemia-induced endothelial damage, and reduction of oxidative stress in retinal tissues [[7], [8], [9]], suggesting a potential molecular basis for its role in preventing diabetes-induced retinal changes.

Optical coherence tomography angiography (OCTA) is a non-invasive imaging modality that enables detailed visualization of the retinal microvasculature, which is widely used to detect vascular alterations associated with various retinal diseases [[10], [11], [12]]. In DR, characteristic OCTA findings—beyond those detectable via fundus examination—include reductions in the vessel length density (VLD), perfusion deficits, foveal avascular zone (FAZ) enlargement, and diminished choriocapillaris flow [13]. Emerging evidence highlights the clinical relevance of deep capillary plexus (DCP) nonperfusion in non-proliferative DR (NPDR), as deeper retinal ischemia has been associated with disease severity and the risk of subsequent complications [14]. Therefore, quantitative OCTA-derived parameters, including VLD and perfusion index (PI), may serve as sensitive biomarkers for detecting early changes in retinal microvascular perfusion in DR.

Recent experimental studies have highlighted the potential role of AMP-activated protein kinase (AMPK)-related signaling pathways in mediating the vascular protective effects of certain ginseng-derived constituents. For example, ginsenoside Ro has been reported to attenuate endothelial injury through Epac1–AMPK–mediated mitochondrial stabilization [15], while arginyl-fructosyl-glucose (AFG) and ginsenoside Rd have been associated with AMPK activation and preservation of endothelial barrier integrity under hyperglycemic conditions [16,17]. Given that endothelial dysfunction, oxidative stress, and metabolic imbalance are central to the pathogenesis of diabetic microangiopathy, these preclinical findings provide a biologically plausible framework for investigating whether KRG may influence retinal microvascular parameters. However, direct clinical evidence linking KRG administration to quantitative OCTA-derived retinal perfusion metrics remains limited.

As the global prevalence of diabetes continues to rise, so has the associated socioeconomic burden [18], underscoring the importance of effective management and treatment strategies for diabetic complications. Therefore, this prospective, randomized, double-blind clinical trial aimed to investigate short-term changes in OCTA-derived retinal microvascular perfusion parameters, with a particular focus on DCP VLD and PI, in patients with mild-to-moderate NPDR following KRG administration.

## 2 Materials and methods

This study aimed to evaluate the changes in central macular thickness (CMT), best-corrected visual acuity (BCVA), and OCTA parameters between patients with DR who received KRG extract as adjunctive therapy and those who did not. The study was approved by the Institutional Review Board of the Yonsei University Health System (IRB approval number: 3-2020-0475).

### 2.1 Study design

This study entailed a single-center, prospective, randomized, double-blind clinical trial. Patients who were clinically diagnosed with mild to moderate NPDR and did not exhibit signs of PDR or DME were followed-up prospectively for 3 months. Single-blind allocation to the test or control groups was achieved using block randomization implemented with Microsoft Excel®. Double-blinding was maintained throughout the study to ensure that both the participants and treating physicians were unaware of the group assignments. The allocation sequence was generated by the Biostatistics team at Gangnam Biomedical Research Center of Yonsei University College of Medicine. Participants were enrolled by a certified Clinical Research Coordinator (CRC), who also verified eligibility criteria and managed all follow-up visits. The CRC then assigned participants to interventions according to the pre-determined allocation sequence. At baseline, systemic demographic variables including age, sex, duration of diabetes, presence of hypertension, hyperlipidemia, history of cardiovascular or cerebrovascular accidents, HbA1c, and systolic and diastolic blood pressure were recorded. At each visit, all participants underwent BCVA testing, intraocular pressure (IOP) measurement, optical coherence tomography (OCT), and OCTA. These tests were performed at baseline for initial data collection, after which patients were administered either KRG extract or placebo for 90 days. Thereafter, changes in the CMT, BCVA, and OCTA parameters —including FAZ, VLD, and PI in the SCP and DCP—were evaluated. The occurrence of complications such as PDR or DME was also assessed.

Participants assigned to the treatment group received KRG tablets (500 mg per tablet; 4 tablets daily, total 2 g/day) for 90 days. The product was manufactured by Korea Ginseng Corporation (Daejeon, Republic of Korea). The standardized extract was derived from 6-year-old *Panax ginseng* roots and formulated as film-coated tablets. The total ginsenoside content was 8 mg per daily dose, quantified based on the marker compounds Rg1, Rb1, and Rg3, in accordance with established manufacturing and quality-control standards for commercial KRG formulations. While minor ginsenosides and other phytochemical constituents may be present in trace amounts inherent to the extract, the product was standardized and quality-controlled using these representative marker compounds.

No formal interim analysis or prespecified early stopping rule was implemented. Recruitment was completed within the predefined study period, and the final sample size reflected logistical and enrollment constraints rather than a data-driven stopping decision.

The primary outcome was the time × group interaction effect for VLD and PI in the DCP between baseline and 3 months, as assessed using participant-clustered generalized estimating equations (GEE). Secondary outcomes included changes in SCP parameters, FAZ area, BCVA, IOP, CMT, and safety outcomes.

### 2.2 Eligibility criteria

Patients diagnosed with mild to moderate NPDR were enrolled in the study. The exclusion criteria were as follows: 1) pregnant or lactating women and individuals under 19 years of age; 2) patients with comorbid ocular diseases that may significantly affect visual acuity or CMT, such as glaucoma, age-related macular degeneration, or branch retinal vein occlusion; 3) patients with a history of ocular trauma or surgery; 4) patients taking medications other than antihypertensive or antidiabetic agents; 5) patients with severe diabetic ocular complications, including PDR, DME, or neovascular glaucoma; and 6) patients taking circulatory-enhancing agents as adjunctive therapy for diabetes. Participants were withdrawn from the study under the following conditions: 1) development of a serious medical condition unrelated to the study, 2) noncompliance with the investigator's instructions, and 3) voluntary withdrawal by the participant or inability to continue participation due to personal circumstances.

### 2.3 Sample size calculation

To estimate the minimum number of participants required for this clinical study, a priori power analysis was performed using G∗Power (v3.1; Heinrich-Heine-Universität Düsseldorf, Düsseldorf, Germany). Assuming an alpha level of 0.05 and a statistical power of 80%, the repeated-measures analysis of variance (ANOVA) was used to evaluate changes between the past and present time points within the same patient group. Assuming two repeated measurements, 34 participants were included in this study. Considering a 10% dropout rate, the study aimed to recruit 38 participants, with 19 patients allocated to the treatment and control groups each.

The a priori sample size calculation was performed at the participant level using a repeated-measures ANOVA framework as a pragmatic design-stage approximation. In the final dataset, because OCTA parameters were analyzed at the eye level and both eyes from the same participant could be included, participant-clustered GEE was applied to account for within-subject correlation. Therefore, the initial ANOVA-based sample size estimation should be interpreted as an approximate planning tool rather than an exact reflection of the final analytic model.

### 2.4 Image acquisition and quantitative analysis

**Fig. 1:**
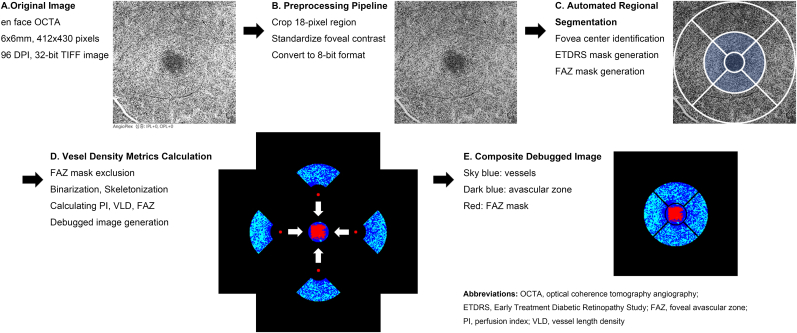
Workflow for regional analysis of en face optical coherence tomography angiography (OCTA) Images. (A) Original image, (B) Preprocessing pipeline, (C) Automated regional segmentation, (D) Vessel density metrics calculation, and (E) Composite debugged image.

The Zeiss Cirrus HD-OCT 5000 AngioPlex system (software version 11.0, A-scan rate: 68,000 scans/s) was used to capture 6 × 6 mm OCTA images composed of 175 B-scans. All OCTA images were reviewed prior to analysis to confirm appropriate layer segmentation and image integrity. The exported image files (412 × 430 pixels, 96 DPI, 32-bit, LZW-compressed TIFF format) were processed using a custom pipeline implemented in Python 3.13.5. A representative en face OCTA image is presented prior to any processing (Fig. 1A).

The image was then subjected to a preprocessing pipeline (Fig. 1B). The bottom 18-pixel region was cropped from each image to remove overlaid text labels that could interfere with the subsequent analysis. To standardize the foveal contrast across images, image brightness was adjusted programmatically using a custom Python script to achieve a median foveal intensity of approximately 45 (8-bit scale). This target value was empirically determined based on visual evaluation of representative cases and remained fixed for all subsequent preprocessing steps.

Automated regional segmentation was performed (Fig. 1C). The foveal center was automatically identified using a hybrid approach that combined connected-component analysis and low-intensity clustering. Based on the detected center, regional masks—visually represented as blue zones in Fig. 1C— were constructed to match the inner macular ring configuration established by the Early Treatment Diabetic Retinopathy Study. The macula was divided into five subfields: a central zone (0.5 mm radius) and four perifoveal quadrants (0.5–1.5 mm annulus), corresponding to the superior, inferior, nasal, and temporal regions.

Vessel density metrics were calculated within the defined regions (Fig. 1D). The FAZ was excluded from the analysis of the central zone to avert confounding effects on VLD and perfusion measurements. The image is then binarized using the Otsu method to separate vessels from the background, followed by skeletonization to measure vessel length. The PI was calculated as the proportion of vessel-positive pixels within each region, whereas the VLD was defined as the total length of the skeletonized vessel map divided by the regional area (mm^2^). Pixel-wise measurements were converted into physical units based on a known scaling factor (6 mm/412 pixels = 0.01456 mm/pixel). All analyses were fully automated, and debugged images were generated to visually verify region segmentation, binarization accuracy, and skeleton quality. The final composite debugged image (Fig. 1E) integrates all subfields and visually summarizes the vessel density analysis. In this image, sky blue represents vessels, dark blue indicates avascular zones, and red highlights the FAZ mask. Fig. 1 illustrates the overall image processing workflow, including representative examples of each step.

This prospective randomized controlled trial was conducted between April 20, 2021, and February 28, 2022, with patient enrollment spanning April to November 2021 and a 3-month follow-up period thereafter. At the time of study design and data acquisition, high-resolution OCTA imaging of the DCP was limited by the available hardware and software.

### 2.5 Outcomes

The primary outcome was the change in the BCVA, CMT, and OCTA parameters, including the FAZ area, VLD, and PI of both the SCP and DCP, between baseline and 3 months. The secondary outcome was the occurrence of adverse events or complications during the treatment period.

### 2.6 Statistical analysis

All statistical analyses were performed using Python (version 3.13.5), with libraries including pandas, NumPy, SciPy, Pingouin, and statsmodels. Statistical significance was defined as p < 0.05.

Descriptive statistics were used to summarize baseline characteristics. Continuous variables were assessed for normality using the Shapiro–Wilk test. Between-group comparisons at baseline were performed using the independent two-sample *t*-test or Mann–Whitney *U* test for continuous variables and the chi-squared test or Fisher's exact test for categorical variables, as appropriate.

Systemic clinical variables (HbA1c, systolic blood pressure[SBP], and diastolic blood pressure[DBP]) were analyzed at the patient level. To evaluate longitudinal changes over time (baseline and 3 months), two-way repeated-measures analysis of variance (ANOVA) was performed to assess the main effects of time and group and the time × group interaction. Given the presence of two time points, the sphericity assumption was inherently satisfied.

Ocular parameters derived from OCTA were analyzed at the eye level. Because randomization was performed at the patient level while measurements were obtained from both eyes in some participants, participant-clustered GEE with an exchangeable working correlation structure and robust (sandwich) standard errors were applied to account for within-participant inter-eye correlation. Fixed effects included group, time, and the group × time interaction term. The primary inferential focus was the group × time interaction.

When significant interaction effects were observed, post hoc within-group time effects were estimated using the same clustered modeling framework. Effect sizes were calculated where appropriate. No correction for multiple comparisons was applied, given the exploratory nature of this pilot study.

## 3 Results

### 3.1 Demographics

**Fig. 2:**
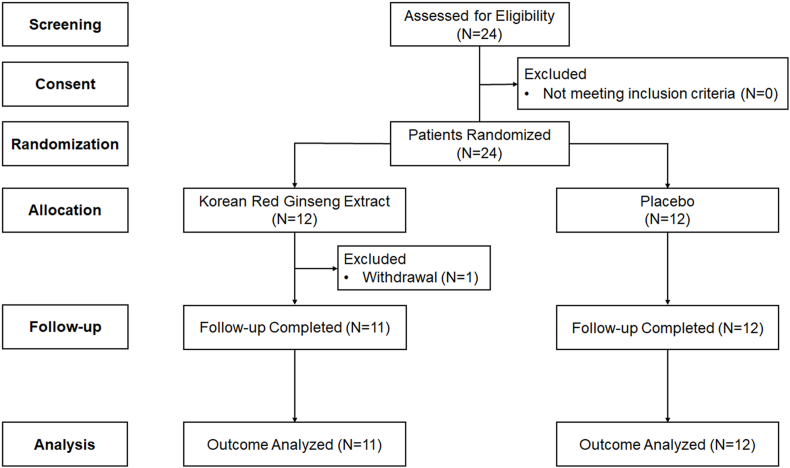
CONSORT flow diagram showing enrollment, randomization, withdrawal, and completion of 90-day treatment and follow-up in 24 patients assigned to Korean Red ginseng extract or placebo.

A total of 24 patients were enrolled in this study. One participant (GSSev-10) withdrew consent during the study period and was excluded from the final analysis. Thus, 23 patients completed the 90-day treatment with either KRG extract or placebo and subsequent follow-up. The overall flow of participants through the trial, including recruitment, randomization, follow-up, and analysis, is illustrated in Fig. 2. The participants’ mean age was 61.09 ± 10.01 years, and 8 (34.8%) were women. The treatment group included 11 patients (22 eyes), whereas the control group included 12 patients (24 eyes). No statistically significant differences in age, sex, presence of hypertension, hyperlipidemia, history of cardiovascular or cerebrovascular accidents, duration of diabetes, HbA1c levels or baseline systolic and diastolic blood pressure were evident between the two groups (all p > 0.05; Table 1).

**Table 1:** Baseline characteristics of patients with diabetic retinopathy stratified by group.

Variable	Treatment group (n = 11)	Control group (n = 12)	P-value
Age (years)	62.64 ± 9.49	59.67 ± 10.88	0.492^a^
Sex (female), n (%)	4 (36.4%)	4 (33.3%)	0.999^b^
Duration of DM (months)	187.45 ± 118.62	163.58 ± 108.15	0.621^a^
HbA1c (%)	7.16 ± 0.41	7.36 ± 0.71	0.536^a^
HTN, n (%)	6 (54.5%)	9 (75.0%)	0.400^b^
SBP (mmHg)	121.36 ± 11.51	126.83 ± 15.36	0.343^a^
DBP (mmHg)	71.73 ± 7.31	76.08 ± 13.32	0.339^a^
DL, n (%)	8 (72.7%)	11 (91.7%)	0.317^b^
CVA, n (%)	2 (18.2%)	2 (16.7%)	0.999^b^

DM, diabetes mellitus; HbA1c, glycated hemoglobin; HTN, hypertension; SBP, systolic blood pressure; DBP, diastolic blood pressure; DL, dyslipidemia; CVA: cerebrovascular or cardiovascular accident, including stroke and/or myocardial infarction.

a Independent *t*-test.

b Fisher's exact test.

### 3.2 Temporal changes in systemic variables between the treatment and control groups

Two-way repeated-measures ANOVA showed no significant time × group interaction effects for systemic parameters, including HbA1c and blood pressure (HbA1c, p = 0.847; SBP, p = 0.545; DBP, p = 0.834; Supplementary Table S3). Neither the main effect of time nor the main effect of group was statistically significant (all p > 0.05). The interaction effect sizes were small (partial η^2^ = 0.002 for HbA1c, 0.018 for SBP, and 0.002 for DBP), indicating minimal differential systemic changes between groups over the 3-month study period.

### 3.3 Changes in best-corrected visual acuity, intraocular pressure, central macular thickness, and optical coherence tomography angiography parameters

The baseline BCVA, IOP, CMT, or OCTA parameters did not differ significantly between the treatment and control groups (all p > 0.05; Supplementary Table S1).

Longitudinal analyses were performed using participant-clustered GEE. The p-values shown in Supplementary Table S2 correspond to the group × time interaction terms derived from these models for BCVA, IOP, and CMT. No statistically significant interaction effects were observed (all p > 0.05), indicating that the temporal changes in these parameters did not differ significantly between the treatment and control groups.

Consistent with these findings, within-group time effects estimated using the same clustered modeling framework (Supplementary Table S4) were not statistically significant (all p > 0.05), further supporting the absence of detectable treatment-related effects on visual acuity or macular structural parameters over the 3-month period. Sensitivity analyses adjusting for baseline HbA1c using participant-clustered GEE models yielded consistent results, with no significant group × time interactions observed (Supplementary Table S7). No progression of DR severity was observed in either group over the 3-month study period.

### 3.4 Temporal changes in superficial capillary plexus vessel length density and perfusion index between the treatment and control groups

The Group × Time interaction for SCP OCTA parameters (VLD and PI across regions, and FAZ area) was evaluated using participant-clustered GEE models. No significant Group × Time interactions were observed for any SCP metrics (all p > 0.05; Table 2), indicating comparable temporal changes between the treatment and control groups over the 3-month follow-up.

**Table 2:** Group × time interaction effects from baseline to 3 Months for superficial capillary plexus parameters estimated using participant-clustered generalized estimating equations.

Outcome	Time Point	Treatment (mean ± SD)	Control (mean ± SD)	Group × Time Interaction (P-value)
VLD – Central (mm^−1^)	Baseline	6.46 ± 2.76	7.44 ± 3.33	0.776
	3 Months	5.88 ± 2.79	7.09 ± 3.13	
VLD – Superior (mm^−1^)	Baseline	15.55 ± 2.49	15.32 ± 2.74	0.297
	3 Months	14.94 ± 2.84	15.51 ± 2.52	
VLD – Inferior (mm^−1^)	Baseline	15.50 ± 2.49	15.46 ± 2.90	0.802
	3 Months	15.28 ± 2.52	15.40 ± 2.05	
VLD – Nasal (mm^−1^)	Baseline	15.50 ± 2.75	16.01 ± 2.64	0.597
	3 Months	15.00 ± 3.06	16.03 ± 2.07	
VLD – Temporal (mm^−1^)	Baseline	15.70 ± 2.46	15.66 ± 2.64	0.570
	3 Months	14.93 ± 2.83	15.30 ± 2.43	
P – Central (%)	Baseline	14.44 ± 6.40	16.79 ± 7.97	0.623
	3 Months	12.88 ± 6.21	16.18 ± 7.61	
P – Superior (%)	Baseline	37.59 ± 6.36	37.04 ± 7.30	0.374
	3 Months	36.16 ± 7.41	37.48 ± 7.08	
P – Inferior (%)	Baseline	37.57 ± 6.78	37.87 ± 7.79	0.708
	3 Months	36.81 ± 6.53	37.75 ± 5.39	
P – Nasal (%)	Baseline	36.93 ± 7.32	38.26 ± 6.94	0.656
	3 Months	35.60 ± 8.01	38.06 ± 5.76	
P – Temporal (%)	Baseline	37.52 ± 6.56	37.56 ± 6.92	0.574
	3 Months	35.75 ± 7.15	36.84 ± 6.39	
FAZ (mm^2^)	Baseline	0.34 ± 0.12	0.29 ± 0.13	0.944
	3 Months	0.32 ± 0.14	0.28 ± 0.12	

P-values correspond to the group × time interaction terms estimated using participant-clustered generalized estimating equations (GEE) with an exchangeable working correlation structure and robust (sandwich) standard errors. Analyses were conducted at the eye level with clustering at the participant level to account for inter-eye correlation.

SD, standard deviation; VLD, vessel length density; P, perfusion index; FAZ, foveal avascular zone.

Consistently, post hoc within-group analyses using the same clustered framework revealed no significant changes from baseline to 3 months in either group (all p > 0.05; Supplementary Table S5). HbA1c-adjusted sensitivity analyses showed consistent findings, with no significant group × time interactions for any SCP parameters (Supplementary Table S7).

### 3.5 Temporal changes in deep capillary plexus vessel length density and perfusion index between the treatment and control groups

**Fig. 3:**
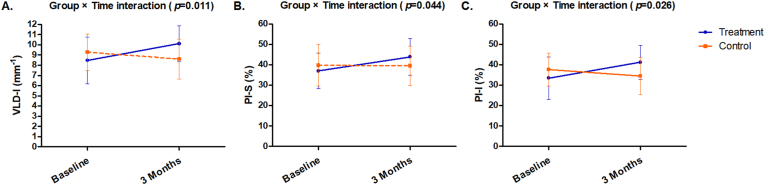
Changes in deep capillary plexus microvascular parameters from baseline to 3 months in treatment and control groups. (A) Vessel length density (VLD)–inferior; (B) Perfusion index (PI)–superior; (C) PI–inferior. Group × Time interaction p-values are shown.

The Group × Time interaction for DCP OCTA parameters was evaluated using participant-clustered GEE models. Significant interaction effects were observed for inferior VLD (VLD-I, p = 0.011), as well as for PI in the superior (PI-S, p = 0.044) and inferior (PI-I, p = 0.026) regions (Table 3). The temporal changes in these parameters are illustrated in Fig. 3. No other DCP subfields demonstrated statistically significant interaction effects.

**Table 3:** Group × time interaction effects from baseline to 3 Months for deep capillary plexus parameters estimated using participant-clustered generalized estimating equations.

Outcome	Time Point	Treatment (mean ± SD)	Control (mean ± SD)	Group × Time Interaction (P-value)
VLD – Central (mm^−1^)	Baseline	2.27 ± 2.45	2.33 ± 1.74	0.100
	3 Months	2.86 ± 2.45	1.95 ± 0.96	
VLD – Superior (mm^−1^)	Baseline	9.10 ± 1.56	9.68 ± 2.10	0.083
	3 Months	10.75 ± 1.86	9.56 ± 1.86	
VLD – Inferior (mm^−1^)	Baseline	8.47 ± 2.30	9.29 ± 1.77	0.011∗
	3 Months	10.12 ± 1.76	8.61 ± 1.96	
VLD – Nasal (mm^−1^)	Baseline	9.31 ± 2.01	9.55 ± 1.91	0.774
	3 Months	10.46 ± 2.29	9.44 ± 2.01	
VLD – Temporal (mm^−1^)	Baseline	8.83 ± 1.76	8.97 ± 1.98	0.224
	3 Months	9.49 ± 2.06	8.44 ± 1.80	
P – Central (%)	Baseline	7.25 ± 9.17	7.04 ± 6.33	0.145
	3 Months	7.79 ± 7.78	5.82 ± 3.70	
P – Superior (%)	Baseline	37.02 ± 8.71	39.80 ± 10.13	0.044∗
	3 Months	43.93 ± 8.98	39.48 ± 9.67	
P – Inferior (%)	Baseline	33.50 ± 10.38	37.73 ± 8.05	0.026∗
	3 Months	41.21 ± 8.41	34.51 ± 9.19	
P – Nasal (%)	Baseline	38.23 ± 9.84	39.91 ± 9.74	0.742
	3 Months	44.17 ± 10.93	39.27 ± 9.08	
P – Temporal (%)	Baseline	36.38 ± 8.49	36.39 ± 9.07	0.809
	3 Months	38.56 ± 9.36	33.67 ± 8.42	
FAZ (mm^2^)	Baseline	0.40 ± 0.05	0.41 ± 0.05	0.476
	3 Months	0.42 ± 0.04	0.41 ± 0.04	

P-values correspond to the group × time interaction terms estimated using participant-clustered generalized estimating equations (GEE) with an exchangeable working correlation structure and robust (sandwich) standard errors. Analyses were conducted at the eye level with clustering at the participant level to account for inter-eye correlation.

SD, standard deviation; VLD, vessel length density; P, perfusion index; FAZ, foveal avascular zone.

∗*p* < 0.05.

Post hoc within-group analyses showed that VLD-I and PI-S/PI-I increased from baseline to 3 months in the treatment group (all p < 0.05), whereas no significant longitudinal changes were detected in the control group (Supplementary Table S6). Between-group comparisons of change scores were directionally consistent with the interaction results.

Collectively, these findings suggest region-specific differences in temporal microvascular changes within the DCP between the treatment and control groups over the 3-month follow-up period. Sensitivity analyses adjusting for baseline HbA1c yielded consistent results, with significant group × time interactions persisting for VLD-I, PI-S, and PI-I (Supplementary Table S7).

### 3.6 Adverse events

During the 3-month observation period, no cases of progression to PDR, development of DME, or IOP elevation were observed among the study participants. In addition, no adverse events related to the intake of KRG extract or placebo were reported.

## 4 Discussion

In this prospective randomized trial, significant group × time interaction effects were observed for inferior DCP vessel length density (VLD-I) and for perfusion index in the superior and inferior DCP regions (PI-S and PI-I). These findings indicate region-specific differences in temporal microvascular changes between the treatment and control groups over the 3-month period. No significant interaction effects were identified for SCP parameters, BCVA, IOP, or CMT. Apart from one participant who withdrew consent, no adverse events were reported. To our knowledge, this is one of the first randomized investigations exploring the short-term effects of KRG extract on OCTA-derived microvascular parameters in patients with NPDR.

Given the lack of differences in baseline characteristics between the groups, the randomization process appears to have been conducted appropriately. The lack of significant changes in the BCVA, IOP, CMT, and most OCTA parameters over the relatively short 3-month period was consistent with the participants’ NPDR status. Nevertheless, previous studies have demonstrated that DCP perfusion tends to decline with increasing DR severity, highlighting its potential as a sensitive marker of disease progression [19]. In this study, no significant changes in DCP or SCP perfusion were observed in the control group during the 3-month follow-up period. Given the limited sample size and inherent variability of OCTA measurements, these findings should be interpreted cautiously and do not necessarily indicate stability or progression of underlying disease. Furthermore, sensitivity analyses adjusting for baseline HbA1c yielded consistent findings, indicating that the results were not materially altered after accounting for baseline glycemic status.

In this study, significant group × time interaction effects were observed for DCP VLD-I and for PI-S and PI-I. These findings indicate region-specific differences in temporal microvascular changes observed in the group receiving KRG compared with controls over the 3-month period. Importantly, no significant time × group interaction effects were observed for systemic parameters, including HbA1c and blood pressure, and the associated effect sizes were negligible. These findings suggest that the observed DCP microvascular changes were unlikely to be driven by differential systemic metabolic or hemodynamic changes between groups.

The KRG extract used in this study was standardized to the major ginsenosides-Rg1, -Rb1, and -Rg3, which have been implicated in vascular and neuroprotective mechanisms in experimental models. Ginsenoside-Rg1 has been shown to inhibit retinal endothelial cell proliferation, migration, and VEGF expression [20], thereby suppressing vascular leakage and capillary degeneration [21]. Ginsenoside-Rb1 has been reported to mitigate high glucose-induced oxidative injury [22], while Rg3 alleviates retinal injury by attenuating ROS-mediated endoplasmic reticulum stress [23].

In the present study, significant group × time interaction effects were observed for DCP VLD-I and PI-S and PI-I, indicating region-specific differences in temporal microvascular changes between the treatment and control groups over the 3-month period. In the context of prior reports highlighting the susceptibility and functional relevance of the DCP in DR, these findings may suggest short-term modulation of deep retinal microvascular parameters in the group receiving KRG. However, given the exploratory nature of this pilot study and the limited follow-up duration, whether such alterations translate into sustained structural preservation or functional benefit remains to be determined.

One of the most critical vascular changes in the progression of DR involves the DCP. Previous studies have demonstrated that the DCP–VLD tends to decrease in the early stages of DR [24,25], and this reduction has been proposed as a predictive marker for complications such as vitreous hemorrhage, macular edema, and progression to PDR [14]. Recent quantitative OCTA studies and meta-analyses have further confirmed that DCP perfusion parameters decline even in mild NPDR and show progressive reduction with increasing disease severity [26]. Notably, VLD reduction is often observed earlier in the DCP than in the SCP [19,27], underscoring the particular vulnerability of the deep retinal microvasculature. Quantitatively, vessel length density derived from OCTA represents the extent of perfused capillary networks within a given retinal slab and is widely used as a surrogate marker of microvascular perfusion status. Because OCTA detects motion contrast generated by flowing erythrocytes and has a limited dynamic range for flow detection, reductions in DCP VLD are generally interpreted as reflecting capillary nonperfusion or perfusion deficit rather than definitively demonstrating structural vessel loss [28]. Consistent with this interpretation, prior studies have demonstrated that quantitative DCP perfusion metrics are associated with diabetic retinopathy severity and visual function [29], correlate with ultrawide-field fluorescein angiography–defined nonperfusion areas [30], and are significantly related to visual acuity and low-luminance vision in diabetic retinopathy [31], underscoring their physiological and clinical relevance. Furthermore, DCP nonperfusion has been shown to correlate strongly with visual function and DR severity [31]. This phenomenon may be attributed to the early loss of pericytes [19], which impairs capillary perfusion, as well as the structural vulnerability of the venous system within the retinal vascular architecture [25]. These findings underscore the importance of monitoring changes in the DCP, which could serve as a sensitive biomarker of disease progression in NPDR.

In contrast to the characteristic VLD decline reported in previous studies, our study did not observe a significant reduction in the DCP–VLD over the 3-month period, which may be attributed to the relatively short observation window and limited sample size. However, significant group × time interaction effects were identified for DCP VLD-I and for PI-S and PI-I, indicating region-specific differences in temporal microvascular changes between the treatment and control groups. Rather than reflecting reversal of established microvascular damage, these findings may suggest short-term modulation of selected deep retinal perfusion parameters in the group receiving KRG. Given the exploratory nature of this pilot study, whether such alterations translate into sustained structural stability, functional preservation, or delayed disease progression warrants further investigation in larger, long-term studies. In addition, although the absolute DCP VLD values in our study were lower than those reported in studies using built-in software—likely due to differences in image processing pipelines—absolute OCTA-derived vascular density values are known to vary substantially across analytic frameworks. Prior work has demonstrated limited agreement between different OCTA analysis programs, particularly between skeletonized and non-skeletonized metrics, even when identical imaging hardware is used [32]. Accordingly, direct comparison of absolute values across studies may be inappropriate. Rather, the relative within-study changes observed under a consistent processing protocol provide the most methodologically interpretable signal in the present exploratory analysis. Nonetheless, these findings should be interpreted as reflecting short-term alterations in microvascular metrics rather than established clinical benefit.

A strength of this study lies in its prospective, randomized, double-blind design and its focus on quantitative OCTA-derived DCP parameters in patients with NPDR. However, some limitations of this study should be acknowledged. First, the final sample size was smaller than initially planned, which may have limited statistical power. Post-hoc power analysis indicated that the achieved statistical power was approximately 63% under the original design assumptions, suggesting reduced sensitivity to detect small-to-moderate effects. Therefore, non-significant findings should be interpreted cautiously, as they may reflect type II error. Accordingly, this study should be regarded as an exploratory pilot randomized trial aimed at detecting early microvascular signals rather than establishing definitive clinical efficacy. Second, the relatively short duration of administration and follow-up (3 months) may have limited the ability to detect sustained or progressive microvascular changes. Third, the single-center design and exclusion of patients with advanced diabetes-related complications limit generalizability to broader NPDR populations. Fourth, VLD measurements using OCTA are highly sensitive to image-processing pipelines, including binarization, skeletonization, and segmentation algorithms. In this study, SCP parameters were analyzed using the in-built AngioPlex software, whereas DCP parameters were extracted using a custom Python-based pipeline owing to the lack of built-in support for DCP segmentation. The absolute values of VLD in our study were lower than previously reported ranges {typically 17–18 mm^−1^ in healthy DCP layers [33,34]}, as measured using Zeiss-provided software such as the Density Exerciser. This discrepancy probably reflects the differences in the image processing pipeline, including the skeletonization method, thresholding, and segmentation criteria. Importantly, because all images in this study were processed using the same parameters and analyzed comparatively, the relative changes within groups are valid and interpretable. Accordingly, this study focused on relative changes from baseline rather than absolute values. To support this approach, Supplementary Table S8 presents a comparison of the VLD values (mm^−1^) acquired using the Cirrus HD-OCT 5000 system and reported in several peer-reviewed studies [[33], [34], [35], [36], [37], [38], [39], [40]], demonstrating substantial variability across studies despite identical hardware. Fifth, the above-mentioned limitations reflect technological constraints at the time of study design and data acquisition (April 2021 to February 2022). These include the use of the Cirrus HD-OCT 5000 system, which captures 175 B-scans over a 6 × 6 mm area at a scan rate of 68,000 A-scans/s, potentially limiting the resolution for reliable DCP segmentation. Future research leveraging newer OCTA platforms and more advanced segmentation algorithms may further refine and validate the findings presented in this study. Finally, because OCTA parameters were analyzed at the eye level while randomization was performed at the participant level, GEE was selected to account for within-participant inter-eye correlation and to provide robust population-level inference. Although GEE does not explicitly model subject-specific random effects, it was considered appropriate given the study design and the primary objective of estimating group-level treatment effects.

## 5 Conclusion

At 3 months, a statistically significant time × group interaction was observed for the VLD and PI in selected DCP regions, indicating greater short-term changes in OCTA-derived microvascular perfusion metrics in the KRG group compared with placebo. These changes were not accompanied by differences in visual acuity, macular thickness, or the development of PDR or DME during the study period.

Given the reduced sample size and short follow-up, the findings should be interpreted cautiously and considered exploratory. Nevertheless, the study demonstrates the feasibility of OCTA in detecting early microvascular dynamics in NPDR.

Future adequately powered, multicenter studies with extended follow-up durations, standardized imaging protocols, and high-resolution OCTA platforms are warranted to validate these findings and to determine whether such microvascular changes translate into meaningful clinical benefit in patients with NPDR.

## Funding

This study was supported by a research grant conferred by the Korean Society of Ginseng’s 2020 Red Ginseng Research Project.

## Conflict of interest statement

The authors declare no conflicts of interest.
