# Supplementary material for: Effect of Korean red ginseng on deep capillary plexus parameters in diabetic retinopathy: A prospective, randomized, double-blind clinical trial
Source: J Ginseng Res. 2026 Mar 31;50(4):101025. doi: 10.1016/j.jgr.2026.101025 (PMC13323880; doi:10.1016/j.jgr.2026.101025)
Supplement: Multimedia component 1 [file mmc1.docx]

**Supplementary Table S1. Baseline Ocular Findings in the Treatment and Control Groups**

| Variable | Treatment group  (mean ± SD) | Control group  (mean ± SD) | P-value |
| --- | --- | --- | --- |
| logMAR BCVA | 0.02 ± 0.04 | 0.02 ± 0.05 | 0.960^†^ |
| IOP (mmHg) | 13.73 ± 3.12 | 13.93 ± 3.59 | 0.839^‡^ |
| CMT (μm) | 251.41 ± 22.55 | 257.00 ± 22.03 | 0.400^‡^ |
| SCP |  |  |  |
| VLD – Central (mm^-1^) | 6.46 ± 2.76 | 7.44 ± 3.33 | 0.285^‡^ |
| VLD – Superior (mm^-1^) | 15.55 ± 2.49 | 15.32 ± 2.74 | 0.895^†^ |
| VLD – Inferior (mm^-1^) | 15.50 ± 2.49 | 15.46 ± 2.90 | 0.904^†^ |
| VLD – Nasal (mm^-1^) | 15.51 ± 2.75 | 16.01 ± 2.64 | 0.441^†^ |
| VLD – Temporal (mm^-1^) | 15.71 ± 2.46 | 15.66 ± 2.64 | 0.951^‡^ |
| P – Central (%) | 14.44 ± 6.40 | 16.79 ± 7.97 | 0.274^‡^ |
| P – Superior (%) | 37.59 ± 6.36 | 37.04 ± 7.30 | 0.999^†^ |
| P – Inferior (%) | 37.57 ± 6.78 | 37.87 ± 7.79 | 0.792^†^ |
| P – Nasal (%) | 36.93 ± 7.32 | 38.26 ± 6.94 | 0.538^†^ |
| P – Temporal (%) | 37.52 ± 6.56 | 37.56 ± 6.92 | 0.986^‡^ |
| FAZ | 0.34 ± 0.12 | 0.29 ± 0.13 | 0.208^‡^ |
| DCP |  |  |  |
| VLD – Central (mm^-1^) | 2.27 ± 2.45 | 2.33 ± 1.74 | 0.531^†^ |
| VLD – Superior (mm^-1^) | 9.10 ± 1.56 | 9.68 ± 2.10 | 0.285^‡^ |
| VLD – Inferior (mm^-1^) | 8.47 ± 2.30 | 9.29 ± 1.77 | 0.186^‡^ |
| VLD – Nasal (mm^-1^) | 9.310 ± 2.01 | 9.55 ± 1.91 | 0.675^‡^ |
| VLD – Temporal (mm^-1^) | 8.83 ± 1.76 | 8.98 ± 1.98 | 0.797^‡^ |
| P – Central (%) | 7.25 ± 9.17 | 7.04 ± 6.33 | 0.700^†^ |
| P – Superior (%) | 37.02 ± 8.71 | 39.80 ± 10.13 | 0.296^†^ |
| P – Inferior (%) | 33.50 ± 10.38 | 37.73 ± 8.05 | 0.133^‡^ |
| P – Nasal (%) | 38.23 ± 9.84 | 39.91 ± 9.74 | 0.564^‡^ |
| P – Temporal (%) | 36.38 ± 8.49 | 36.39 ± 9.07 | 0.997^‡^ |
| FAZ (mm²) | 0.40 ± 0.05 | 0.41 ± 0.05 | 0.851^†^ |

SD, standard deviation; logMAR, logarithm of the minimum angle of resolution; BCVA, best-corrected visual acuity; IOP, intraocular pressure; CMT, central macular thickness; VLD, vessel length density; P, perfusion index; FAZ, foveal avascular zone

^†^Mann–Whitney *U* test

^‡^Independent *t*-test

**Supplementary Table S2. Group × Time Interaction Effects from Baseline to 3 Months for Best-Corrected Visual Acuity, Intraocular Pressure, and Central Macular Thickness Estimated Using Participant-Clustered Generalized Estimating Equations**

| Outcome | Time Point | Treatment  (mean ± SD) | Control  (mean ± SD) | Group × Time Interaction  (P-value) |
| --- | --- | --- | --- | --- |
| logMAR BCVA | Baseline | 0.02 ± 0.04 | 0.02 ± 0.05 | 0.198 |
|  | 3 Months | 0.02 ± 0.05 | 0.01 ± 0.03 |  |
| IOP (mmHg) | Baseline | 13.73 ± 3.12 | 13.93 ± 3.59 | 0.454 |
|  | 3 Months | 13.91 ± 2.31 | 15.08 ± 2.96 |  |
| CMT (μm) | Baseline | 251.41 ± 22.55 | 257.00 ± 22.03 | 0.125 |
|  | 3 Months | 250.64 ± 22.14 | 258.54 ± 21.76 |  |

P-values correspond to the group × time interaction terms estimated using participant-clustered generalized estimating equations (GEE) with an exchangeable working correlation structure and robust (sandwich) standard errors. Analyses were conducted at the eye level with clustering at the participant level to account for inter-eye correlation.

SD, standard deviation; logMAR, logarithm of the minimum angle of resolution; BCVA, best-corrected visual acuity; IOP, intraocular pressure; CMT, central macular thickness

**Supplementary Table S3. Two-way Repeated-Measures ANOVA Assessing Time, Group, and Time × Group Interaction Effects for Systemic Clinical Variables**

| Outcome | N (T/C) | ∆ Treatment (mean ± SD) | ∆ Control (mean ± SD) | P (Time) | P (Group) | P (Time × Group) | Partial η² |
| --- | --- | --- | --- | --- | --- | --- | --- |
| HbA1c (%) | 11 / 12 | -0.02 ± 0.47 | 0.02 ± 0.58 | 0.969^†^ | 0.060^†^ | 0.847^†^ | 0.002 |
| SBP (mmHg) | 11 / 12 | −2.09 ± 12.99 | 0.75 ± 8.99 | 0.795^†^ | 0.148^†^ | 0.545^†^ | 0.018 |
| DBP (mmHg) | 11 / 12 | −1.18 ± 5.79 | −0.58 ± 7.56 | 0.545^†^ | 0.471^†^ | 0.834^†^ | 0.002 |

∆ = 3 months - baseline

T, treatment; C, control; SBP, systolic blood pressure; DBP, diastolic blood pressure

^†^Two-way Repeated Measures ANOVA

**Supplementary Table S4. Post Hoc within-Group Time Effects from Baseline to 3 Months for Visual Acuity, Intraocular Pressure, and Central Macular Thickness Estimated Using Participant-Clustered Generalized Estimating Equations**

| Outcome | Group | β | SE | 95% CI | P-value |
| --- | --- | --- | --- | --- | --- |
| logMAR BCVA | Treatment | 0.005 | 0.010 | -0.015 to 0.024 | 0.652 |
|  | Control | -0.013 | 0.009 | -0.029 to 0.004 | 0.146 |
| IOP (mmHg) | Treatment | 0.182 | 0.941 | -1.662 to 2.026 | 0.847 |
|  | Control | 1.154 | 0.895 | -0.599 to 2.907 | 0.197 |
| CMT (μm) | Treatment | -0.773 | 1.161 | -3.049 to 1.503 | 0.506 |
|  | Control | 1.542 | 0.960 | -0.340 to 3.423 | 0.108 |

Analyses were performed using participant-clustered generalized estimating equations (GEE) with an exchangeable working correlation structure and robust (sandwich) standard errors. β represents the estimated within-group time effect from baseline to 3 months.

SE, standard error; CI, confidence interval; logMAR, logarithm of the minimum angle of resolution; BCVA, best-corrected visual acuity; IOP, intraocular pressure; CMT, central macular thickness

**Supplementary Table S5. Post Hoc within-Group Time Effects from Baseline to 3 Months in Superficial Capillary Plexus Parameters Estimated Using Participant-Clustered Generalized Estimating Equations**

| Outcome | Group | β | SE | 95% CI | P-value |
| --- | --- | --- | --- | --- | --- |
| VLD – Central (mm^-1^) | Treatment | -0.582 | 0.699 | -1.952 to 0.789 | 0.405 |
|  | Control | -0.350 | -0.416 | -1.166 to 0.466 | 0.400 |
| VLD – Superior (mm^-1^) | Treatment | -0.614 | 0.681 | -1.948 to 0.721 | 0.368 |
|  | Control | 0.192 | 0.362 | -0.519 to 0.902 | 0.597 |
| VLD – Inferior (mm^-1^) | Treatment | -0.214 | 0.439 | -1.075 to 0.648 | 0.627 |
|  | Control | -0.054 | 0.459 | -0.954 to 0.845 | 0.906 |
| VLD – Nasal (mm^-1^) | Treatment | -0.500 | 0.851 | -2.168 to 1.168 | 0.557 |
|  | Control | 0.025 | 0.510 | -0.974 to 1.024 | 0.961 |
| VLD – Temporal (mm^-1^) | Treatment | -0.773 | 0.608 | -1.965 to 0.419 | 0.204 |
|  | Control | -0.362 | 0.390 | -1.128 to 0.403 | 0.353 |
| P – Central (%) | Treatment | -1.559 | 1.642 | -4.777 to 1.659 | 0.342 |
|  | Control | -0.613 | 1.010 | -2.591 to 1.366 | 0.544 |
| P – Superior (%) | Treatment | -1.427 | 1.865 | -5.083 to 2.229 | 0.444 |
|  | Control | 0.437 | 0.961 | -1.446 to 2.321 | 0.649 |
| P – Inferior (%) | Treatment | -0.759 | 1.190 | -3.091 to 1.572 | 0.523 |
|  | Control | -0.117 | 1.233 | -2.533 to 2.299 | 0.925 |
| P – Nasal (%) | Treatment | -1.327 | 2.116 | -5.475 to 2.820 | 0.531 |
|  | Control | -0.200 | 1.391 | -2.927 to 2.527 | 0.886 |
| P – Temporal (%) | Treatment | -1.768 | 1.581 | -4.868 to 1.331 | 0.264 |
|  | Control | -0.717 | 0.997 | -2.671 to 1.238 | 0.472 |
| FAZ (mm²) | Treatment | -0.013 | 0.026 | -0.065 to 0.039 | 0.618 |
|  | Control | -0.015 | 0.018 | -0.051 to 0.020 | 0.390 |

Analyses were performed using participant-clustered generalized estimating equations (GEE) with an exchangeable working correlation structure and robust (sandwich) standard errors. β represents the estimated within-group time effect from baseline to 3 months.

SE, standard error; CI, confidence interval; VLD, vessel length density; P, perfusion index; FAZ, foveal avascular zone

**Supplementary Table S6. Post Hoc within-Group Time Effects from Baseline to 3 Months in Deep Capillary Plexus Parameters Estimated Using Participant-Clustered Generalized Estimating Equations**

| Outcome | Group | β | SE | 95% CI | P-value |
| --- | --- | --- | --- | --- | --- |
| VLD – Central (mm^-1^) | Treatment | 0.543 | 0.433 | -0.305 to 1.391 | 0.209 |
|  | Control | -0.397 | 0.374 | -1.129 to 0.336 | 0.289 |
| VLD – Superior (mm^-1^) | Treatment | 0.658 | 0.353 | -0.034 to 1.349 | 0.062 |
|  | Control | -0.183 | 0.332 | -0.833 to 0.46 | 0.581 |
| VLD – Inferior (mm^-1^) | Treatment | 0.699 | 0.228 | 0.252 to 1.146 | 0.002^*^ |
|  | Control | -0.279 | 0.309 | -0.885 to 0.326 | 0.366 |
| VLD – Nasal (mm^-1^) | Treatment | 0.181 | 0.338 | -0.481 to 0.843 | 0.592 |
|  | Control | 0.047 | 0.322 | -0.583 to 0.678 | 0.884 |
| VLD – Temporal (mm^-1^) | Treatment | 0.383 | 0.375 | -0.351 to 1.117 | 0.306 |
|  | Control | -0.195 | 0.293 | -0.769 to 0.378 | 0.504 |
| P – Central (%) | Treatment | 2.473 | 2.829 | -3.071 to 8.017 | 0.382 |
|  | Control | -2.635 | 2.073 | -6.697 to 1.428 | 0.204 |
| P – Superior (%) | Treatment | 2.404 | 2.052 | -1.618 to 6.425 | 0.241 |
|  | Control | -2.949 | 1.681 | -6.243 to 0.346 | 0.079 |
| P – Inferior (%) | Treatment | 2.955 | 1.483 | 0.048 to 5.861 | 0.046^*^ |
|  | Control | -2.379 | 1.884 | -6.072 to 1.314 | 0.207 |
| P – Nasal (%) | Treatment | 0.058 | 1.834 | -3.536 to 3.652 | 0.975 |
|  | Control | -0.721 | 1.503 | -3.666 to 2.224 | 0.631 |
| P – Temporal (%) | Treatment | -0.775 | 2.797 | -6.258 to 4.707 | 0.782 |
|  | Control | -1.563 | 1.685 | -4.865 to 1.739 | 0.354 |
| FAZ (mm²) | Treatment | -0.035 | 0.022 | -0.079 to 0.008 | 0.107 |
|  | Control | -0.016 | 0.017 | -0.048 to 0.017 | 0.338 |

Analyses were performed using participant-clustered generalized estimating equations (GEE) with an exchangeable working correlation structure and robust (sandwich) standard errors. β represents the estimated within-group time effect from baseline to 3 months.

SE, standard error; CI, confidence interval; VLD, vessel length density; P, perfusion index; FAZ, foveal avascular zone

^*^*p*<0.05

**Supplementary Table S7. Sensitivity Analysis of Group × Time Interaction Effects Adjusted for Baseline HbA1c Using Generalized Estimating Equations Across Ocular Parameters and OCTA Metrics (Superficial and Deep Capillary Plexus).**

| Domain | Outcome | β (Group × Time) | SE | 95% CI | P-value |
| --- | --- | --- | --- | --- | --- |
| Ocular | logMAR BCVA | 0.017 | 0.013 | -0.009 to 0.043 | 0.198 |
|  | IOP (mmHg) | -0.972 | 1.298 | -3.517 to 1.572 | 0.454 |
|  | CMT (μm) | -2.314 | 1.507 | -5.268 to 0.639 | 0.125 |
| SCP | VLD – Central (mm^-1^) | -0.232 | 0.814 | -1.827 to 1.363 | 0.776 |
|  | VLD – Superior (mm^-1^) | -0.805 | 0.771 | -2.317 to 0.707 | 0.297 |
|  | VLD – Inferior (mm^-1^) | -0.159 | 0.635 | -1.405 to 1.086 | 0.802 |
|  | VLD – Nasal (mm^-1^) | -0.525 | 0.992 | -2.469 to 1.419 | 0.597 |
|  | VLD – Temporal (mm^-1^) | -0.410 | 0.723 | -1.827 to 1.006 | 0.570 |
|  | P – Central (%) | -0.947 | 1.927 | -4.724 to 2.831 | 0.623 |
|  | P – Superior (%) | -1.865 | 2.098 | -5.977 to 2.248 | 0.374 |
|  | P – Inferior (%) | -0.642 | 1.713 | -4.000 to 2.715 | 0.708 |
|  | P – Nasal (%) | -1.127 | 2.533 | -6.091 to 3.836 | 0.656 |
|  | P – Temporal (%) | -1.052 | 1.870 | -4.716 to 2.613 | 0.574 |
|  | FAZ (mm²) | 0.002 | 0.032 | -0.060 to 0.065 | 0.944 |
| DCP | VLD – Central (mm^-1^) | 0.940 | 0.572 | -0.181 to 2.061 | 0.100 |
|  | VLD – Superior (mm^-1^) | 0.841 | 0.484 | -0.109 to 1.790 | 0.083 |
|  | VLD – Inferior (mm^-1^) | 0.978 | 0.384 | 0.226 to 1.731 | 0.011^*^ |
|  | VLD – Nasal (mm^-1^) | 0.134 | 0.466 | -0.780 to 1.048 | 0.774 |
|  | VLD – Temporal (mm^-1^) | 0.579 | 0.475 | -0.353 to 1.510 | 0.224 |
|  | P – Central (%) | 5.107 | 3.507 | -1.766 to 11.981 | 0.145 |
|  | P – Superior (%) | 5.352 | 2.652 | 0.154 to 10.551 | 0.044^*^ |
|  | P – Inferior (%) | 5.334 | 2.398 | 0.634 to 10.034 | 0.026^*^ |
|  | P – Nasal (%) | 0.779 | 2.371 | -3.868 to 5.426 | 0.742 |
|  | P – Temporal (%) | 0.787 | 3.265 | -5.613 to 7.188 | 0.809 |
|  | FAZ (mm²) | -0.020 | 0.028 | -0.074 to 0.034 | 0.476 |

P-values correspond to the Group × Time interaction terms estimated using generalized estimating equations (GEE), adjusted for baseline HbA1c and clustered at the participant level to account for within-participant correlation (including correlation between eyes). An exchangeable working correlation structure was assumed, and robust (sandwich) standard errors were used.

SE, standard error; CI, confidence interval; logMAR, logarithm of the minimum angle of resolution; BCVA, best-corrected visual acuity; IOP, intraocular pressure; CMT, central macular thickness; VLD, vessel length density; P, perfusion index; FAZ, foveal avascular zone

^*^*p*<0.05

**Supplementary Table S8. Reported Deep Capillary Plexus Vessel Density or Vessel Length Density (mm^-1^ or mm/mm²) from Studies Using the Zeiss Cirrus HD‑OCT 5000**

| Study | Device/SW | Scan Size | Scan Speed | Number of B-scans | Analysis Target | Inner Macula Ring Analysis | VLD or Other Metrics | Software Used |
| --- | --- | --- | --- | --- | --- | --- | --- | --- |
| Bujor et al. [35] 2024 | Cirrus HD-5000/AngioPlex (FastTrac) | 3×3 mm | 68,000 A-scans/s | 245×245, 4 B-scan/location | SCP, DCP, CC | No | Perfusion Density (DCP, %)  39.1 ± 3.6 (Chinese) vs. 40.1 ± 3.4 (Caucasian) | AngioPlex /  MATLAB |
| Coppe et al. [36], 2021 | Cirrus HD-5000/AngioPlex | 6×6 mm | 68,000 A-scans/s | 128 B-scan × 512 A-scan | ETDRS grid, SCP, DCP | Yes | Percentage VD (DCP, %)  0.237 ± 0.072 (FEs)  0.255 ± 0251 (CEs) | AngioPlex  PLEX Elite 9000 v1.5.0.15909 |
| Wang et al. [37] 2019 | Cirrus HD-5000 | 3×3 mm | 68,000 A-scans/s | NR | SCP | No | Vessel area density (DCP, %)  27.5 ± 5.5 (F) vs. 45.1 ± 1.8 (T) vs. 46.6 ± 1.8 (S) vs. 45.9 ± 1.9 (I) vs. 45.8 ± 1.8 (N) | MATLAB |
| Inooka et al. [33], 2018 | Cirrus HD‑5000/AngioPlex | 3×3 mm | NR | NR | SCP, DCP | No | VLD (DCP, mm^-1^): 18.448 ± 1.769 (Ctrl) vs. 14.766 ± 2.711 (RP) | AngioPlex / Angio Exerciser |
| Marques et al. [34], 2021 | Cirrus HD‑5000 | 3×3 mm | NR | 245×245, 4 B-scan/location | SCP, DCP | Yes | VLD (DCP, mm^-1^): 17.0 ± 2.14 (CEs) vs. 16.4 ± 2.2 (ETDRS 43-47) | Carl Zeiss Meditec Density Exerciser v10.0.12787 |
| Carnevali et al. [38] 2017 | Cirrus HD‑5000/AngioPlex | 3×3 mm | 68,000 A-scans/s | 245×245, 4 B-scan/location | SCP, DCP | No | VD (%): 0.464 ± 0.016 (DR) vs. 0.477 ± 0.014 (CEs) | ImageJ |
| Lei et al. [39] 2018 | Cirrus HD‑5000 / AngioPlex | NR | 68,000 A-scans/s | NR | Between two concentric circles (1.5 and 2.25mm in diameter) | Yes (partially) | VLD (mm^-1^): 16.9 ± 1.9 (S) vs. 20.7 ± 2.4 (N) vs. 16.8 ± 2.2 (I) vs. 19.8 ± 4.4 (T) | AngioPlex / ImageJ |
| Choi et al. [40] 2020 | Cirrus HD‑5000/AngioPlex | 6×6 mm | 68,000 A-scans/s | 245×245 | SCP, DCP | No | VLD (mm^-1^): 25.43 ± 1.96 vs. 25.32 ± 3.38 | ImageJ v1.50 |

SW, software; SCP, superficial capillary plexus; DCP, deep capillary plexus; VLD, vessel length density; CC, choriocapillary; VD, vessel density; FE, fellow eye of the eye with an epiretinal membrane; CE, control eye; F, fovea; T, temporal; S, superior; I, inferior; N, nasal; RP, retinitis pigmentosa; ETDRS, Early Treatment of Diabetic Retinopathy Study; DR, diabetic retinopathy
